# Lipogenic gene expression profile in patients with gastric cancer

**DOI:** 10.3892/mco.2013.148

**Published:** 2013-07-17

**Authors:** KAZUHITO MIYACHI, YOUKI SAWADA, YOSUKE SHIDA, AKIRA SUGAWARA, HISASHI HISATOMI

**Affiliations:** 1 Department of Surgery, Nikko Medical Center, Dokkyo Medical University, Shimotsuga-gun, Tochigi, 321-0293, Japan; 2 Department of Materials and Life Science, Seikei University, Musashino, Tokyo 180-8633, Japan

**Keywords:** carnitine *O*-palmitoyltransferase type I, miR-33b, sterol regulatory element-binding protein-1, gastric cancer

## Abstract

Numerous types of cancer exhibit increased lipogenesis and expression of lipogenic enzymes and transcription factors, including sterol regulatory element-binding protein-1. Lipogenic gene expression is upregulated at the mRNA level, in concert with metabolic pathways associated with changes in expression and/or activity of lipogenic transcription factors. However, this expression pattern in human gastric carcinoma has not been elucidated. In this study, lipogenic gene expression in cancer tissues was investigated using quantitative PCR. In patients with gastric cancer, *carnitine O-palmitoyltransferase type I* mRNA and *miR-33b* were significantly downregulated, suggesting that *miR-33b* downregulation is mediated by conditions that also affect the expression and/or activity of transcription factors involved in lipogenic gene expression. Consequently, the association between *miR-33b* and gastric cancer may provide a novel strategy for the genetic diagnosis of gastric cancer. However, additional studies including a larger number of samples are required to confirm these results.

## Introduction

Lipogenic genes exert their biological effects through transcriptional regulation of their target genes, several of which are key regulatory genes involved in lipogenic metabolism (1). Sterol regulatory element-binding protein (SREBP)-1c is a key regulator of fatty acid metabolism and plays a pivotal role in the transcriptional regulation of different lipogenic genes that mediate lipid synthesis (2,3). Nuclear SREBP-1c preferentially binds to E-box motifs, thus enhancing transcription of genes required for saturated and unsaturated fatty acid and triglyceride biosynthesis. During this process, the lipogenic mRNAs for ATP citrate lyase (*ACLY*) and fatty acid synthase (*FASN*) are elevated (4,5), whereas the lipogenic mRNAs for carnitine *O*-palmitoyltransferase type I (*CPT-I*) are suppressed by SREBP-1c expression (6,7).

Cancer tissue proliferates by actively using the energy supplied by fatty acid metabolism. In cancer as well as normal cells, the upregulation of lipogenic enzymes is indispensable for fatty acid metabolism and high lipogenic gene expression is typical of a wide variety of cancers (8,9). SREBP-1c is a key transcription factor that affects cholesterol/lipid biosynthesis and uptake. *miR-33b* is embedded in *SREBP-1* introns and targets several key regulators of cholesterol trafficking and of fatty acid/triglyceride homeostasis for post-transcriptional repression (10–12).

Although the mechanisms that underlie lipogenic gene overexpression in gastric cancer have not been elucidated, part of the lipogenic pathway is intensely expressed in metaplasia and in a subset of gastric adenocarcinomas that are characterized by disease progression, tumor aggressiveness and poor patient survival (13,14).

The aim of the present study was to investigate lipogenic gene expression in cancer and non-cancer tissues from gastric cancer patients using quantitative PCR (qPCR) analysis.

## Materials and methods

### mRNA quantification

Samples were obtained during surgical resection of gastric tissues from 34 Japanese gastric cancer patients (22 male and 12 female; median age, 69.6±10.5 years). Cancer and non-cancer tissues were investigated. Non-cancer tissue was sampled at >5 cm from the edge of each gastric cancer nodule. Samples were frozen in RNase Later (Ambion, Foster City, CA, USA) immediately after surgical resection and stored at −80°C until analysis. Written informed consent was obtained from each patient.

Total RNA was extracted from 10 mg of tissue using the Isogen II reagent (Nippon Gene, Tokyo, Japan) according to the manufacturer’s instructions. Complementary DNA (cDNA) was prepared by incubating DNase-treated total RNA (0.1 μg) with PrimeScript^®^ II First Strand cDNA Synthesis kit (Takara Bio, Inc., Shiga, Japan). The qPCR reaction mixture was prepared using FastStart TaqMan^®^ Probe Master (Rox) (Roche Applied Science, Mannheim, Germany) or Kapa Sybr^®^ Fast qPCR Master mix (Kapa Biosystems, Inc., Woburn, MA, USA). Primers for amplifying the *ACLY*, *FASN*, *CPT-I* and *SREBP-1* mRNAs and for *miR-33b*, are presented in Table I.

PCR reactions comprised 45 cycles (at 95°C for 20 sec, at 60°C for 30 sec and at 72°C for 20 sec) with the CFX96 real-time PCR Detection system (Bio-Rad, Foster City, CA, USA). The first PCR reaction comprised 7 cycles (at 95°C for 10 sec, at 60°C for 15 sec and at 72°C for 10 sec) using FastStart TaqMan Probe Master (Rox) (Roche Applied Science). The second reaction comprised 45 cycles (at 95°C for 10 sec, at 60°C for 15 sec and at 72°C for 10 sec) using the CFX96 real-time PCR Detection system (Bio-Rad). The RNA samples were quantified by relating the PCR threshold cycles obtained from the cell line samples to the amplicon-specific standard curves. As normalization to the *GAPDH* housekeeping gene was inaccurate, the RNA expression levels were presented as the mRNA copy number per μg total RNA.

### Statistical analysis

The samples in the experiments were tested in triplicate or quadruplicate. Data are expressed as means ± standard deviation. Differences between the mean values were evaluated using the Student’s t-test. P<0.05 was considered to indicate a statistically significant difference.

## Results

**Figure 1:**
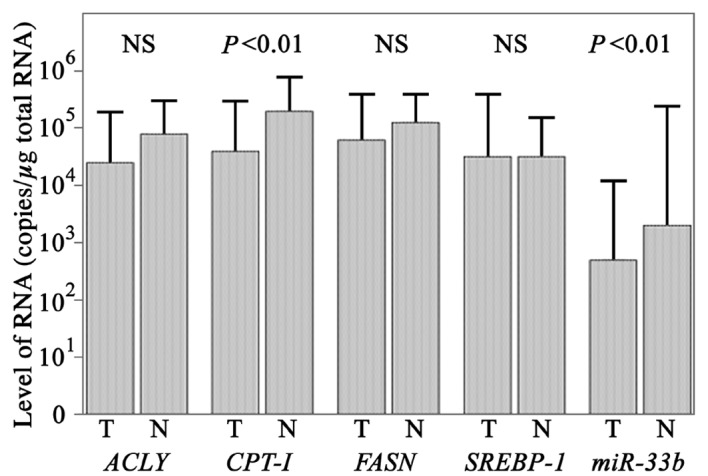
Expression of lipogenic genes in patients with gastric cancer. T, tumor; N, non-tumor. qPCR analyses were performed as described in Materials and methods. RNA expression levels are presented as the number of RNA copies/μg of total RNA.

The levels of CPT-1 mRNA in cancerous tissues were determined as 10^4.6^±10^0.9^ copies/μg total RNA using qPCR (Fig. 1) and were significantly higher in non-cancer tissues at 10^5.3^±10^0.6^ copies/μg total RNA (Student’s t-test; P<0.01). The levels of *miR-33b* in cancerous tissues were determined as 10^2.7^±10^1.4^ copies/μg total RNA and were significantly higher in non-cancerous tissues at 10^3.3^±10^2.1^ copies/μg total RNA (Student’s t-test; P<0.01).

The levels of *SREBP-1* mRNA in cancer and non-cancer tissues were determined as 10^4.5^±10^1.1^ and and 10^4.5^±10^0.7^ copies/μg total RNA, respectively (Fig. 1), using qPCR.

The levels of *ACLY* mRNA in cancer and non-cancer tissues were determined as 10^4.4^±10^0.9^ and 10^4.9^±10^0.6^ copies/μg total RNA, respectively, while the levels of *FASN* mRNA in cancer and non-cancer tissues were determined as 10^4.8^±10^0.8^ and 10^5.1^±10^0.5^ copies/μg total RNA, respectively. The differences for *SREBP-1*, *ACLY* and *FASN* were not statistically significant according to the Student’s t-test.

## Discussion

Although lipogenesis is negligible in the majority of non-malignant adult tissues (15,16), it is upregulated in several tumors, rendering the investigation of endogenous lipogenesis a novel target for the prevention and/or treatment of cancer. This limited-size study demonstrated no statistically significant differences between the levels of mRNA for lipogenic genes and other clinicopathological characteristics, such as tumor size, degree of differentiation, tumor location, stage TNM and p53 mutation. However, *CPT-1* mRNA and *miR-33b* expression were downregulated in the cancer tissues, suggesting that the downregulation of *CPT-1* mRNA may be part of the mechanism responsible for SREBP upregulation and concords with the increased lipogenesis and lipogenic enzyme expression exhibited by a wide variety of cancers.

*miR-33b* mediates the transcription of its target genes, several of which are critical to lipogenesis and cholesterol metabolism (17,18), including *SREBP-1c*, *ACLY* and *FASN*, which increase fatty acid and triglyceride production (17,18). SREBP-1 is synthesized as an integral protein of endoplasmic reticulum membranes. At the nuclear level, mature SREBP-1 activates genes that encode FAS and other lipogenic enzymes by interacting with sterol response elements present in their promoter regions (19). Consistent with this hypothesis, SREBP expression was markedly stimulated by the inhibition of *miR-33b* expression, which may also result in increased fatty acid oxidation and reduced accumulation of fat in the liver stores. Considering the promising results of the use of anti-miRs in preclinical studies, *miR-33b* may become a viable therapeutic target in the future.

Our results provide a basis for more detailed studies on the regulation of SREBP activity and may assist in further investigations of *miR-33b* as a target of gastric cancer treatment. Although the reason for *miR-33b* downregulation in cancerous tissues is uncertain, the elucidation of its association with lipogenic genes may provide insight into gastric carcinogenesis and lead to the development of novel strategies for the genetic diagnosis of gastric cancer.

## Figures and Tables

**Table I:** Sequences of primers and probes.

Gene (primer/probe)	Sequence	Product size (bp)
*ACLY*		
Forward	5′-GCTTGTTGGCGTGGATGAGAA-3′	138
Reverse	5′-ACTCCATCTTTGGTCACTACAA-3′	
Probe	5′-ACACCTGTTGGTCCACGCCCCTGA-3′	
*CPT-I*		
Forward	5′-CGGCCGATGTTACGACAGGT-3′	190
Reverse	5′-GATGTCGCCTTTGCAGTGCC-3′	
Probe	5′-ACTCCTGGGCAGATGCGCCGATCG-3′	
*FASN*		
Forward	5′-GTGTTTGAGGATGTGGTGCTGC-3′	185
Reverse	5′-CTTTCCGGGTGGTCGAAGAG-3′	
Probe	5′-TGTCAGAGAACGGCAACCTGGTAGTGAGTG-3′	
*SREBP-1*		
Forward	5′-GGTAGGAGCCATGGATTGCACTT-3′	164
Reverse	5′-GAGGTGGAGACAAGCTGCCTG-3′	
*miR-33b*		
Forward	5′-TGTCAGGCAACCGTATTCACC-3′	74
1st reverse	5′-CATGCAGTGAGTTAGATGTAGAACGTGCATTGCTGT-3′	
2nd reverse	5′-CATGCAGTGAGTTAGATGTAGAACGTG-3′	
Probe	5′-CTGTTGCATTGCACCACTCACGG-3′	

*ACLY*, ATP citrate lyase; *CPT-I*, carnitine *O*-palmitoyltransferase type I; *FASN*, fatty acid synthase; *SREBP-1*, sterol regulatory element-binding protein 1.

## References

[1] Li YY (2012). Genetic and epigenetic variants influencing the development of nonalcoholic fatty liver disease. World J Gastroenterol.

[2] Yokoyama C, Wang X, Briggs MR (1993). SREBP-1, a basic-helix-loop-helix-leucine zipper protein that controls transcription of the low density lipoprotein receptor gene. Cell.

[3] Jeon TI, Osborne TF (2012). SREBPs: metabolic integrators in physiology and metabolism. Trends Endocrinol Metab.

[4] Lewis CA, Griffiths B, Santos CR (2011). Genetic ablation of S6-kinase does not prevent processing of SREBP1. Adv Enzyme Regul.

[5] Rudolph MC, Monks J, Burns V (2010). Sterol regulatory element binding protein and dietary lipid regulation of fatty acid synthesis in the mammary epithelium. Am J Physiol Endocrinol Metab.

[6] Matthews KA, Ozdemir C, Rawson RB (2010). Activation of sterol regulatory element binding proteins in the absence of Scap in *Drosophila melanogaster*. Genetics.

[7] Sampath H, Miyazaki M, Dobrzyn A (2007). Stearoyl-CoA desaturase-1 mediates the pro-lipogenic effects of dietary saturated fat. J Biol Chem.

[8] Israël M, Schwartz L (2010). The metabolic advantage of tumor cells. Mol Cancer.

[9] Swinnen JV, Brusselmans K, Verhoeven G (2006). Increased lipogenesis in cancer cells: new players, novel targets. Curr Opin Clin Nutr Metab Care.

[10] Horie T, Ono K, Horiguchi M (2010). MicroRNA-33 encoded by an intron of sterol regulatory element-binding protein 2 (Srebp2) regulates HDL in vivo. Proc Natl Acad Sci USA.

[11] Dávalos A, Goedeke L, Smibert P (2011). miR-33a/b contribute to the regulation of fatty acid metabolism and insulin signaling. Proc Natl Acad Sci USA.

[12] Rayner KJ, Esau CC, Hussain FN (2011). Inhibition of miR-33a/b in non-human primates raises plasma HDL and lowers VLDL triglycerides. Nature.

[13] Perez-Tilve D, Heppner K, Kirchner H (2011). Ghrelin-induced adiposity is independent of orexigenic effects. FASEB J.

[14] Menendez JA, Lupu R (2007). Fatty acid synthase and the lipogenic phenotype in cancer pathogenesis. Nat Rev Cancer.

[15] Weiss L, Hoffmann GE, Schreiber R (1986). Fatty-acid biosynthesis in man, a pathway of minor importance. Purification, optimal assay conditions, and organ distribution of fatty-acid synthase. Biol Chem Hoppe Seyler.

[16] Kusakabe T, Maeda M, Hoshi N (2000). Fatty acid synthase is expressed mainly in adult hormone-sensitive cells or cells with high lipid metabolism and in proliferating fetal cells. J Histochem Cytochem.

[17] Najafi-Shoushtari SH, Kristo F, Li Y (2010). MicroRNA-33 and the SREBP host genes cooperate to control cholesterol homeostasis. Science.

[18] Ramírez CM, Goedeke L, Fernández-Hernando C (2011). ‘Micromanaging’ metabolic syndrome. Cell Cycle.

[19] Nohturfft A, DeBose-Boyd RA, Scheek S (1999). Sterols regulate cycling of SREBP cleavage-activating protein (SCAP) between endoplasmic reticulum and Golgi. Proc Natl Acad Sci USA.

